# Effectiveness of transcutaneous tibial nerve stimulation on overactive bladder treatment

**DOI:** 10.12669/pjms.41.6.11658

**Published:** 2025-06

**Authors:** Huseyin Aydogmus, Mustafa Sengul, Ozlem Bolel, Terlan Selin Kotan

**Affiliations:** 1 Huseyin Aydogmus Department of Gynecology and Obstetrics, Izmir Ataturk Research and Training Hospital, Izmir, Turkiye; 2 Mustafa Sengul School of Medicine, Department of Gynecology and Obstetrics, Izmir Katip Celebi University, Turkiye; 3 Ozlem Bolel Department of Physical Medicine and Rehabilitation, Izmir Ataturk Research and Training Hospital, Izmir, Turkiye; 4 Terlan Selin Kotan Department of Gynecology and Obstetrics, Izmir Ataturk Research and Training Hospital, Izmir, Turkiye

**Keywords:** Overactive Bladder, Quality of Life, Tibial Nerve Stimulation

## Abstract

**Objective:**

There is no consensus on the effectiveness, the sites of application and, optimal dosage and frequency of attendance of transcutaneous tibial nerve stimulation (tTNS) on the treatment of overactive bladder. The aim of the study was to evaluate the long-term effectiveness of eight weeks lasting transcutaneous tibial nerve stimulation (TNS) treatment and its effects on the quality of life of the patients.

**Method:**

In this cross-sectional study conducted between September 2022 and December 2023 at a tertiary care center, 56 patients with overactive bladder were allocated to receive transcutaneous tibial nerve stimulation or sham therapy weekly for eight weeks; 30 of them had transcutaneous tibial nerve stimulation (study group) and remaining 26 patients formed the control group. Overactive Bladder Questionnaire Awareness tool V8 (OAB-v8), International Consultation on Incontinence Questionnaire-Urinary Incontinence Short Form (ICIQ-UI SF), and Visual Analog scale (VAS) scales were used for evaluation of bothersome degree. Patients who received tTNS therapy have been evaluated at 3rd and 6th and 12th months and time-dependent change in the Incontinence Quality of Life Questionnaire (I-QOL) scores were determined.

**Results:**

After eight weeks of tibial nerve stimulation treatment, a statistically significant increase was found in the OAB -V8, ICI-Q and VAS scores compared to the sham therapy group (p<0,05). Minimum 50% increase was achieved in I-QOL scores at the 3rd and 6th months of treatment, but the improvement was less than 50% at the12th month.

**Conclusions:**

Transcutaneous TNS is an effective treatment option for women with OAB, but its long-term effectiveness is insufficient. Therefore, rescue sessions to be held at 6-8 month intervals can be used to increase patient satisfaction.

## INTRODUCTION

Overactive bladder (OAB) is defined as urgency (±incontinence), usually with frequency and nocturia in the absence of an underlying pathology.^1^,^2^ The frequency of OAB are the female population is between 12.8-16.9%.^3^,^4^ It is known that social, psychological, occupational, physical, and sexual lives of individuals with OAB are significantly restricted, and their quality of life is adversely affected.^4^-^7^ A stepwise treatment approach is adopted in treatment of OAB, starting with lifestyle changes (i.e. quitting smoking, diet modifications) and following with minimally invasive treatment options. For patients who do not respond to second-line treatments (i.e. anti-muscarinic drugs and beta-mimetics), or who cannot tolerate the side effects, third-line treatment approaches such as peripheral nerve stimulation, botulinum toxin injection, or neuromodulation are applied.^7^,^8^

The tibial nerve is a mixed nerve originating from the L5-S3 roots, which are the same root that the parasympathetic pathway (S2-S4) of the bladder. Tibial nerve stimulation aims to create a neural response by stimulating the peripheral parts of micturition reflexes. It has been determined that direct stimulation of the tibial nerve can inhibit S2-S3 afferents and thus reduce detrusor hyperactivity. Tibial nerve stimulation by using this mechanism, is a simple, minimally invasive, well tolerated technique that is accepted as a conservative and effective treatment for patients.^9^ Tibial nerve stimulation can be performed by both percutaneous tibial nerve stimulation (pTNS) form with needle electrodes directly inserted into the posterior tibial nerve and also transcutaneous tibial nerve stimulation (tTNS) form using surface electrodes over the skin above the nerve. Transcutaneous TNS has advantages such as being easy to apply in office conditions with the patient being dressed, portable and painless.^10^

The majority of studies reported that tTNS was applied in the treatment of OAB for 12 weeks. Especially in cases where the application is performed in healthcare institutions, a twelve-week treatment process may adversely affect patient compliance. There are few studies which compared the six and eight weeks duration of tTNS treatments with placebo and combined therapy in the literature.^10^-^12^ The main goal of the study is assessment of effectiveness of eight weeks lasting tTNS treatment and its effects on the health-relted quality of life.

## METHODS

This is a retrospective cross-sectional study which aimed to determine the effectiveness of eight weeks lasting tTNS treatment and its effects on the quality of life of the patients by using incontinence-related quality of life scales. This study was conducted between September 2022 and December 2023 at a tertiary care center.

### Ethical Approval:

The study was approved by the Institutional Review Board of Izmir Katip Celebi University Medical School (IRB 0150-2023, date: April 27, 2023) and conducted in accordance with the principles of the Declaration of Helsinki. All subjects gave informed voluntary consent before any treatment modality was initiated.

The women with the complaint of urinary incontinence and were diagnosed with idiopathic OAB, who were older than 18 years of age, who could communicate verbally and in writing, who did not respond to the previous treatments or who did not tolerate anticholinergic agents were included the study. Pregnant women, those with diagnosed acute urinary tract infection or those with a diagnosis of gynecological malignancy, uncontrolled diabetes, renal dysfunction, and pelvic organ prolapse higher than stage-II, women who received intravesical botox treatment in the last six months and those with a permanent pacemaker, women diagnosed with neurogenic incontinence were excluded from the study. Patients were not allowed to use anticholinergic or beta mimetic drugs during tTNS treatment. Demographic characteristics of the patients are shown in Table-I.

**Table-I:** Demographic characteristics of the participants.

		tTNS Group n= 27	Sham tTNS Group n=26	p-value
Age (years)	30-39	2 (7.4)	3 (11.5)	0.19
	40-49	4 (14.8)	5 (19.2)	
	50-59	5 (18.5)	6 (23.07)	
	60-69	9 (33.4)	8 (30.76)	
	70+	7 (25.9)	4 (15.38)	
Parity	0	2 (7.4)	3 (11.5)	0.40
	1	6 (22.2)	4 (15.38)	
	2	14 (51.9)	16 (61.53)	
	3	4 (14.8)	2 (7.69)	
	4+	1 (3.7)	1 (3.84)	
Smoking	Smoker	3 (11.1)	4 (15.38)	0.19
	Non smoker	24 (88.9)	22 (84.62)	
BMI kg/m^2^	18-24.9	8 (29.6)	6 (23.07)	0.85
	25-29.9	10 (37)	11 (42.30)	
	30+	9 (33.49)	9 (34.63)	
Past surgical history	No	10 (37)	11 (42.30)	0.41
	Yes	17 (63)	15 (57.69)	
Operation type	Cesarean section	9 (52.99)	10 (38.46)	0.74
	Gynecologic operation	3 (17.6)	2 (7.69)	
	Other surgery	5 (29.4)	3 (11.5)	
Previous antimuscarinic using	Two or more agent	12 (44.4)	13 (50)	0.48
	One agent	10 (37.1)	9 (34.63)	
	None	5 (18.5)	4 (15.38)	

*p <0.05, calculated by Chi-square test. Data are number (percentage).

**Table-III:** Alternation of I-QOL scores over time in tTNS Group.

Mean I-QOL Scores of Patients		Mean (SD)	Percentage change (%)	pvalue
	Pre-treatment	42.1 (10.9)	100	-
	3rd month	85.7 (9.8)	103.2	0.001
	6th month	68.5 (11.8)	63	0.001
	12th month	49.8 (13.3)	18.5	0.51

*p <0.05, calculated by Wilcoxon matched pair signed-rank test.

The study data were collected by using Overactive Bladder Questionnaire Awareness tool V8 (OAB-v8), International Consultation on Incontinence Questionnaire-Urinary Incontinence Short Form (ICIQ-UI SF), Incontinence Quality of Life Questionnaire (I-QOL) and Visual Analog scale (VAS). Moreover, daily fluid intake, urinary frequency, attacks of nocturia, and type and frequency of incontinence of all participants were determined by a three days bladder diary. International Consultation on Incontinence Questionnaire-Urinary Incontinence Short Form (ICIQ SF) was developed by Avery et al. to evaluate the effects of urinary incontinence on quality of life.^13^ Turkish validity and reliability were established by Çetinel et al.^14^ The scale is available in four dimensions. The first three dimensions are scored in the evaluation. The total score for UI severity ranges between 0 and 21 (Appendix-1).

A low score indicates little effect on quality of life, while a high score indicates severe effect. Overactive Bladder Questionnaire Awareness tool (OAB-V8) is developed to raise diagnostic awareness for OAB which is without the gold standard diagnostic test. Urgency, nocturia and urge incontinence are scored between 0 and 5 (0: not at all; 5: very much) according to the frequency and severity of symptoms (Appendix-2). The highest score is 40.^15^ In the original version, cut-off value for diagnosis was eight but in the Turkish validity and reliability study the value was optimized as eleven.^16^ Incontinence Quality of Life Questionnaire (I-QOL) was developed by Wagner et al. to determine the quality of life of patients with urinary incontinence.^17^

**Fig.1:**
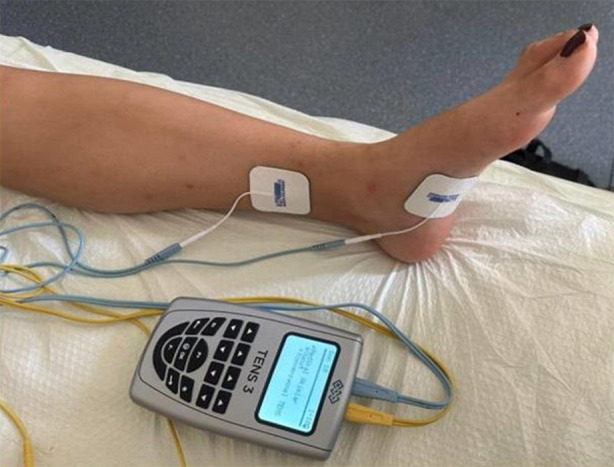
Application of transcutaneous tibial nerve stimulation.

After the Turkish validity and reliability, it was used for the first time by Özerdoğan.^18^ I-QOL consists of three subdomains that limitation of behaviors, psychosocial influence, and social isolation. High scores indicate better quality of life (Appendix-3). Participants were also asked to describe the negative effects of urinary incontinence on their lives on a 1-10 VAS scale. Transcutaneous Tibial Nerve Stimulation (tTNS) was applied to all patients (both of the groups) by a specialist physiotherapist (OB). Two superficial self-adhesive electrodes with 3cm diameter were placed 3 cm cranially of the medial malleolus of one leg, at the level of the tibial bone border, and the other on the plantar surface of the arch of the foot on the same side (Fig.1).

For the study group, a 9-volt alternating current with a frequency of 20 Hz and a pulse duration of 200 ms was applied using a transcutaneous nerve stimulation device (BTL Industries Limited, Hertfordshire, GB) for 30 minutes. Correct placement of the electrodes was geared according to the flexion of the ipsilateral great toe. The intensity of stimulation was determined according to the comfort level of the person. However, in the sham group, the self-adhesive electrodes were inserted as before and connected to the device but without any current. The procedure was repeated once a week for eight weeks.

Our primary endpoint was to compare improvement in the urinary incontinence-related quality of life scores, and the secondary outcome measure was the persistence of a minimum 50% increase in the I-QOL scores over time. An improvement of 50% or more in I-QOL score was accepted as a treatment success. The patients were invited to the clinic at 3rd, 6th and 12th months of the treatment, and the IQOL questionnaire was reapplied, and the change was evaluated according to the pre-treatment values.

SPSS 25.0 software (SPSS® version 25, SPSS, IBM Corporation, NY, USA) was used for statistical analysis. The sample size was calculated as 22 cases to yield 80% power to detect a difference of 2.00 points, assuming an SD of 2.50 points and α-error of 0.05. So in considering the drop rate, the sample size was determined as 27 cases. For the descriptive statistics of the data, the number, percentage, minimum-maximum, values were used. The normal distribution of data belonging to numerical variables was assessed using the Shapiro Wilk normality test. Numerical variables according to groups were compared using the independent samples t test if the data were normally distributed, and the Mann-Whitney U test if the data were not normally distributed. Yates chi-square test, Fisher-Freeman-Halton exact test and Fisher’s exact test were used to compare groups with categorical variables.

The Wilcoxon matched pair signed-rank test was used to compare the treatment success rates of the groups. For evaluation of the early results of the treatment, the results of the two groups, namely real treatment and sham treatment groups, were compared. Also, pre-treatment I-QOL scores of the patients in the real treatment group were compared with the scores at 3, 6 and 12 months after treatment for evaluating the long-term efficacy of the treatment P < 0.05 indicated statistical significance.

## RESULTS

**Fig.2:**
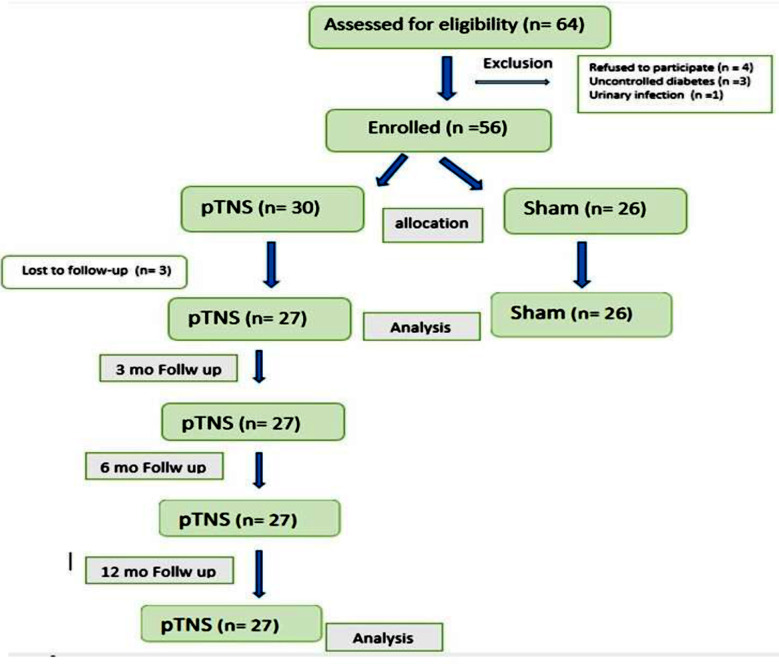
Flow diagram.

In the study period, out of 63 patients who defined urgency incontinence and met the study criteria were included in the study. Eight of them excluded from the study because of several reasons (four patients refused to participate in the study, three patients had uncontrolled diabetes and one patient had urinary tract infection). Remaining 56 participants randomised to the tTNS therapy group (n= 30) and the sham therapy group (n=26). Three patients were excluded from the study group because they did not attend their therapy stage visits (Fig.2).

The groups were found to be similar in terms of age, parity, body mass index, smoking, previous surgical history and previous antimuscarinic using (Table-I). It was determined that the average OAB scores at the end of the treatment were 14.2 in the tTNS group and 26,6 in the sham therapy group, respectively. Average ICIQ-SF score 7.4 in the tTNS group and 14.4 in the sham therapy group, respectively. Average VAS score 4.2 in the tTNS group and 8,4 in the sham therapy group, respectively. There were significant differences between the groups in terms of VAS, OAB-V8, and ICIQ-SF measurements after treatment (*P*<0.05). Table-II summarized the average post-treatment VAS, OAB-V8, and ICIQ-SF scores of the groups.

**Table-II:** Comparison of post treatment quality of life scores of the groups.

		mean(SD)	Median	Min-Max	P value
OAB-V8 Score	Sham	26.6 (6.1)	27	14-40	0.001
	tTNS	14.2 (5.4)	14	5-26	
ICIQ- SF score	Sham	14.4 (3.7)	15	7-21	0.001
	tTNS	7.4 (3.4)	7	1-16	
VAS score	Sham	8.4 (1.5)	9	5-10	0.003
	tTNS	4.2 (1.8)	4	0-7	

*p <0.05, calculated by Wilcoxon matched pair signed-rank test. [Dependent Sample T, Wilcoxon Signed Rank and Mixed Design Repeated Measures Ancova (Pillai’s Trace statistic in the case where the assumption of sphericity is provided)]

The I-QOL questionnaire was used to evaluate the time-dependent change in the quality of life index in patients treated with tTNS. I-QOL scores were improved after treatment compared to pre-treatment levels at three to 12th months. Minimum 50% increase was achieved in I-QOL scores in the 3rd and 6th months of treatment, but the improvement was less than 50% at 12th month (Table-II). Furthermore, by using the 4th dimension of the ICI-Q scale (Appendix 1) it was determined that urgency symptom was the most persistent symptom type after treatment. It was also determined that, the frequency of nocturia, post-void incontinence and unnoticed urinary incontinence symptoms were decreased. According to the bladder diary records, average daily frequency, nocturia and urgency episodes were decreased 65,7%; 70,8% and 69,2%, respectively.

## DISCUSSION

It was determined in the present study that symptom severity decreased, and incontinence-related quality of life scores improved significantly in women with OAB syndrome after eight weeks of tTNS treatment comparing the sham therapy group. It was also determined that incontinence dependent quality of life improves during the first six months after treatment then decreases over time. In the 12th month of treatment, it has been found that the quality of life scores was at a level close to the pre-treatment period.

It has shown in the several studies that transcutaneous tibial nerve stimulation is effective in the treatment of idiopathic OAB and that it improves the objective and subjective parameters of the patients.^12^,^19^,^20^ In a very recent study, Shah et al. reported that the improvement in OAB symptoms with transcutaneous tibial nerve stimulation was not significant compared to sham treatment.^21^ In our study, the difference between tTNS and sham treatment was significant. We think that this difference between the results is due to the treatment application methods. Because in the study conducted by Shah et al., tTNS was applied by the patient himself at home, but in our study, tTNS was applied by a physiotherapist.

In comparative studies with percutaneous tibial nerve stimulation (pTNS), it has been reported that tTNS is comparable to pTNS in reducing the frequency of daily micturition and increasing the quality of life.^22^,^23^ Superioriorities of tTNS to pTNS that it is an almost non-invasive treatment option due to the use of superficial electrodes. Also, adverse events such as pain, bleeding, and discomfort are not observed in the application area. Zhang et al reported that 12 weeks of low-frequency tTNS treatment resulted in a significant reduction in urinary urgency symptoms compared to sham treatment and no significant adverse effects were observed. However, they concluded the improvement in other OAB symptoms such as nocturia was insufficient and that combined treatment applications may be beneficial.^24^

Furthermore, due to its ease of use, it provides the opportunity for patients to perform self-administration without having to visit to a health institution.^25^ Practise differences such as duration of treatment with tTNS (6-12 weeks) and sessions once or twice a week have been reported in the literature.^26^ Self- administrations of tTNS treatment at home is one of the important factors that increase patient compliance. It has been reported that the rate of discontinuation of treatment can increase up to 70%, even in home self-treatment practices.^25^

We found that the quality of life scores were at the maximum level in the 3rd month following the tTNS treatment, and the score decreased slightly in the 6th month, but it was still more than 50% of the initial value. But at the 12th month, the score decreased below 50%. This finding is consistent with the literature. The rate of improvement in OAB symptoms with tTNS treatment has been reported 71.6% - 83.3% in the first three months.^27^ However, studies have reported that the effectiveness of tTNS treatment decreased as time passed.^25^,^27^

In a study which is evaluating the long-term effects of tTNS treatment in urinary incontinence with the longest follow-up period in the literature, Schreiner et al. reported a decrease in urge incontinence episodes in 80.5% of the patients in 12 months follow-up.^28^ In our study, we found 65,7% to 70,8% improvement in daily OAB symptoms in early period and also an increase of 50% or more in quality of life scores in the 3rd and 6th months. But in the 12th month, the increase in quality of life scores were less than 50%.

There is no standard practice in studies evaluating the efficacy of tTNS in the treatment of urinary incontinence. Galhardo et al showed that unilateral and bilateral TTNS applications were equally effective in reducing OAB symptoms and improving the quality of life of patients.^29^ It has been reported that in various methods such as unilateral-bilateral electrode application, initial treatment for 6, 8, 12 weeks, and self-treatment at home with an ambulatory device after basic training, early results are generally successful.^27^ In our study, after eight weeks of treatment, patients were followed up to 12 months, and it was determined that initially improved their quality of life scores regressed up to a point to the baseline values at the 12th month.

We determined that 8-week TTNS treatment (instead of 12-week) significantly increased OAB-related quality of life, but the treatment effectiveness decreased after the 6th month. So, 6 to 8 weeks re-treatment sessions every 6-12 months, may provide long-term symptom relief for patients with minimal side effects. Further studies are needed to investigate whether the repeated application of tTNS treatment can contribute on long-term recovery, and the effectiveness of shorter treatment sessions.

### Strengths & Limitations:

The strengths of our study are that tTNS efficacy was evaluated with more than one QoL scale and it had a relatively long follow-up period. The limitation of the study is evaluation the patients without performing urodynamic studies. Nevertheless, we believe that our study has remarkable results in regulating tTNS treatment programs in terms of duration and repeatability.

## CONCLUSION

The effectiveness, ease of use and safety of tTNS for OAB symptoms makes it considerable as a first-line treatment option along with other conservative methods, especially in early and mid-term period.

### Author’s Contribution:

**HA and MS:** Study design.

**TSK:** Literature search and drafting manuscript.

**HA and OB:** Data collection and analysis.

**HA and MS:** Manuscript writing.

**HA:** Critical revision of the article.

**HA** is responsible and accountable for the accuracy or integrity of the work.

## Figures and Tables

**Appendix:**
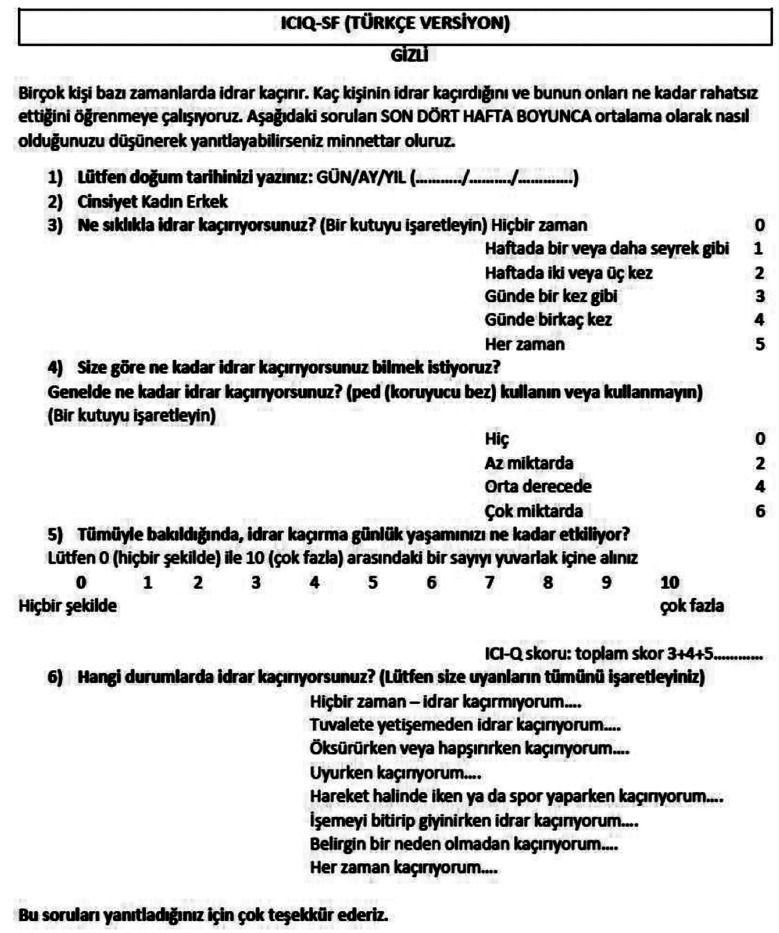
The Turkish version of ICIQ-SF.

**Appendix:**
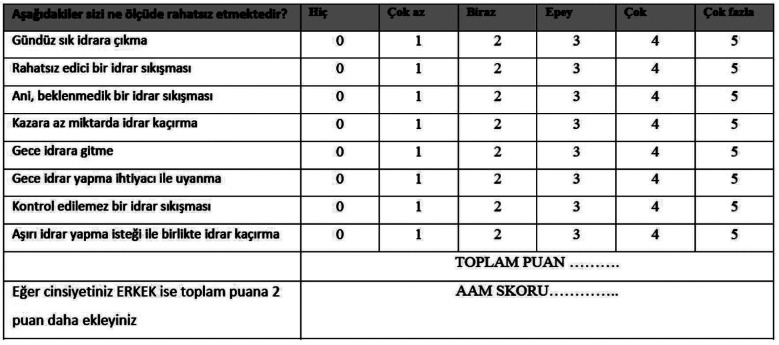
Turkish version of OAB-V8.

**Appendix:**
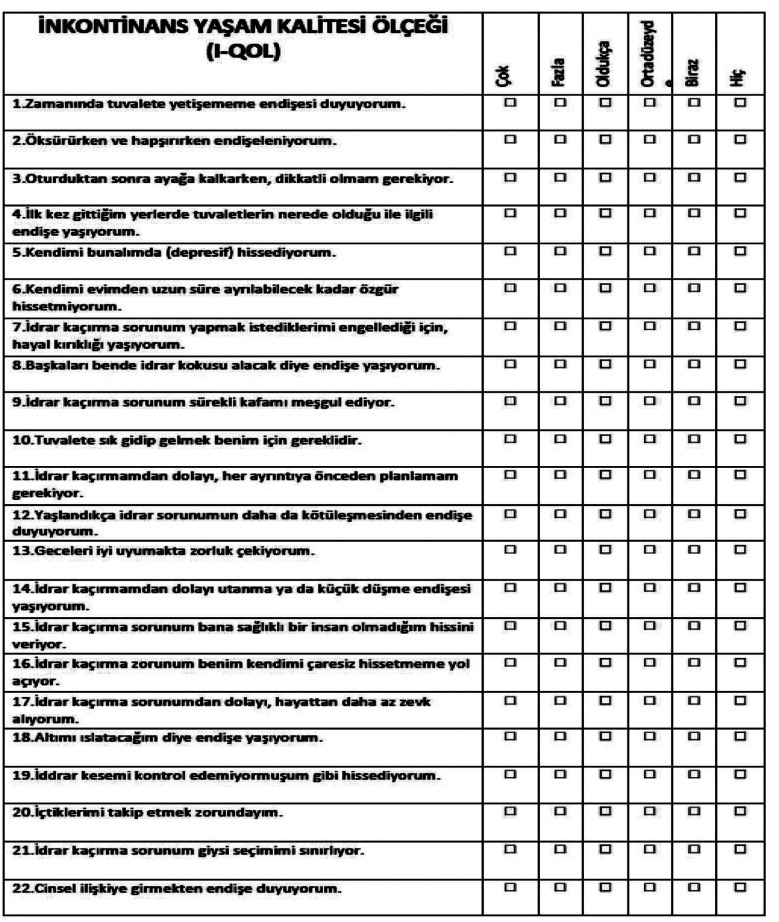
Turkish version of I-QOL.
